# Population aging and trends of pulmonary tuberculosis incidence in the elderly

**DOI:** 10.1186/s12879-021-05994-z

**Published:** 2021-03-25

**Authors:** Shi-jin Li, Yi-fan Li, Wan-mei Song, Qian-yun Zhang, Si-qi Liu, Ting-ting Xu, Qi-qi An, Jin-yue Liu, Huai-chen Li

**Affiliations:** 1 Department of Respiratory and Critical Care Medicine, Shandong Provincial Hospital Affiliated to Shandong University, Shandong Provincial Hospital Affiliated to Shandong First Medical University, 324 Jingwuweiqi Road, Huaiyin District, 250021 Jinan, Shandong People’s Republic of China; 2 Cheeloo College of Medicine, Shandong University, 250012 Jinan, Shandong People’s Republic of China; 3 Department of Intensive Care Unit, Shandong Provincial Third Hospital, 100191 Jinan, Shandong People’s Republic of China; 4 College of Traditional Chinese Medicine, Shandong University of Traditional Chinese Medicine, 16369 Jingshi Road, Lixia District, 250355 Jinan, Shandong People’s Republic of China

**Keywords:** Aging, Pulmonary tuberculosis, Incidence, Join-point regression model

## Abstract

**Background:**

To explore population aging and the epidemic trend of pulmonary tuberculosis (PTB) in the elderly, and provide a basis for the prevention and control of pulmonary tuberculosis among the elderly.

**Methods:**

We collected clinical information of 239,707 newly active PTB patients in Shandong Province from 2005 to 2017. We analyzed and compared the clinical characteristics, reported incidence and temporal trend of PTB among the elderly group (≥60 years) and the non-elderly group (< 60 years) through logistic model and Join-point regression model.

**Results:**

Among the total PTB cases, 77,192(32.2%) were elderly. Compared with non-elderly patients, newly active elderly PTB patients account for a greater proportion of male cases (OR 1.688, 95% CI 1.656–1.722), rural population cases (OR 3.411, 95% CI 3.320–3.505) and bacteriologically confirmed PTB cases (OR 1.213, 95%CI 1.193–1.234). The annual reported incidence of total, elderly, pulmonary bacteriologically confirmed cases were 35.21, 68.84, 35.63 (per 100,000), respectively. The annual reported incidence of PTB in the whole population, the elderly group and the non-elderly group has shown a slow downward trend since 2008. The joinpoint regression model showed that the overall reported incidence of PTB in the elderly significantly decreased from 2007 to 2017 (APC = -5.3, *P* < 0.05). The reported incidence of bacteriologically confirmed PTB among elderly patients declined rapidly from 2005 to 2014(2005–2010 APC = -7.2%, *P* < 0.05; 2010–2014 APC = -22.6%, *P* < 0.05; 2014–2017 APC = -9.0%, *P* = 0.1). The reported incidence of clinically diagnosed PTB among elderly patients from 2005 to 2017 (11.48–38.42/100,000) increased by about 235%. It rose significantly from 2007 to 2014 (APC = 9.4, *P*<0.05).

**Conclusions:**

Compared with the non-elderly population, the reported incidence of PTB in the elderly population is higher. The main burden of PTB will shift to the elderly, men, rural population, and clinically diagnosed patients. With the intensification of aging, more researches on elderly PTB prevention and treatment will facilitate the realization of the global tuberculosis (TB) control targets.

## Background

The World Health Organization (WHO) estimated that 10 million people worldwide suffered from tuberculosis in 2018, of which 1.4 million died. Recent years, the global burden of PTB has been relatively stable. PTB accounted for 85% of all notified tuberculosis cases worldwide, and 88% of deaths from tuberculosis [1]. According to the data from the fifth national tuberculosis epidemiological sampling survey in China in 2010, there were still 4.99 million active PTB patients nationwide [2]. Although the number and incidence of TB in China had been declining in recent years, there were still 833,000 newly diagnosed tuberculosis cases in 2019, with an incidence rate of 58/100,000. Among them, PTB formed a sizeable majority (about 95%) [3]. Globally, the number of new cases of tuberculosis in China still ranked second in 2018, accounting for 9% of all new cases worldwide [1].

Elderly people are both susceptible to new TB infection, and at high risk for reactivation of latent TB. So the elderly population represents a large reservoir of TB infection [4]. Elderly PTB patients have a low positive rate of sputum smears, making diagnosis difficult and more likely to have delayed diagnosis. In addition, in the elderly, due to decreased immunity, more chronic comorbidities and more prone to treatment-related adverse drug reactions, the treatment effect is poor and the mortality rate is high [4–7]. Therefore, with the increase of the aging population, PTB is still one of the diseases that cannot be ignored. The prevention and control of PTB in the elderly need more attention.

China’s aging process is accelerating and it entered an aging society in 1999. The Report on the Development of China’s Elderly Career (2013) issued by the Chinese Academy of Social Sciences pointed out that elderly population in china has exceeded 200 million, and will increase by 1 million per year by 2025 [8]. Shandong Province is the most populous province in China. Shandong’s elderly population ranks first in the country by scale. In 2017, Shandong Province had a population of 23.173 million people aged 60 and above, accounting for 21.4% of the total population of the province. Moreover, the aging population of Shandong Province is experiencing a period of fast development [9], and the aging population of Shandong Province is significantly higher than that of the whole country.

This article describes and compares the reported incidence and trends of newly active PTB among elderly (≥60 years) and non-elderly (< 60 years) in seven cities in Shandong Province, China from 2005 to 2017.

## Methods

### Ethics statement

Ethical approvals of this study were obtained from the Ethics Committee of Shandong Provincial Hospital, affiliated with Shandong University (SPH) and the Ethic Committee of Shandong Provincial Chest Hospital (SPCH), China. Before data analysis and reporting, all personal identifiers of TB patients were removed.

### Study population and data collection

In this study, 77,192 elderly and 162,515 non-elderly new PTB cases were collected from the PTB information management system of the Shandong Center for Disease Control and Prevention (CDC). PTB must be reported within 24 h and registered in the CDC system. Failure to report is a crime in China. Because the reporting and registration of PTB are mandatory in China within the law, CDC has a very lower missing error rate of data on PTB incidence, the data can largely reflect the actual incidence. This study investigated the reported PTB cases in 7 cities in Shandong Province (Dezhou, Jinan, Jining, Liaocheng, Linyi, Weifang and Yantai) from 2005 to 2017. It covered 54% of the population, 50% of health institutions and 51% of health stations in Shandong Province. This study collected data on demographics, clinical information, and disease incidence. The Shandong Statistical Yearbook provided population data every year.

### Laboratory methods and laboratory quality control

All patients with presumptive PTB (cough or fever for more than 2 weeks, weight loss or dysplasia, history of tuberculosis exposure, abnormal chest radiographs) were required to submit at least 2 sputum specimens and use the Ziehl-Neelsen smear microscope to check for acid-fast bacilli (AFB) before starting treatment. Sputum specimens were collected through expectoration, gastric suction, induced sputum and bronchoscopy. For internal quality control, all positive smears were reconfirmed by another examiner in the same laboratory. For external quality assessment, 10% of the isolates were randomly selected from each laboratory and blindly inspected by the upper-level laboratory.

### Data inclusion and definitions

The diagnostic criteria for bacteriologically confirmed PTB was at least 2 smear-positive sputum specimens, or 1 smear-positive sputum specimen plus chest radiograph abnormalities consistent with active PTB, or 1 smear-positive sputum specimens plus 1 culture-positive sputum specimen. The clinically diagnosis of PTB mainly depended on clinical symptoms (cough, fever, hemoptysis, etc.), abnormal chest radiographs, pathology, TST, anti-tuberculosis treatment effects, etc. Except for HIV co-infected patients (in China, HIV-positive people are immediately referred to an HIV specialist hospital), all PTB cases were included in this study. The new case criteria were patients who had never received tuberculosis treatment or had taken anti-tuberculosis drugs for < 1 month. Patients who were diagnosed as active after tuberculosis cure or treatment (whether it is a real relapse or a new episode of tuberculosis caused by reinfection) were relapsed cases.

### Statistical analyses

Categorical variables including gender, race, class, geographic location, patient type (bacteriologically confirmed PTB or clinically diagnosed PTB) were summarized with proportions; continuous variables were summarized with average values. We calculated the annual reported incidence rate (100,000) by dividing the annual number of cases by the annual population size. We used the method to calculate the total reported incidence rate and calculate the reported incidence rate classified by gender, age group, patient type and geographic location. Through univariate analysis, odds ratios (ORs) and 95% confidence intervals (CIs) were obtained. Chi-square test was used to compare the specificity of elderly and non-elderly PTB patients in some aspects, and *p* < 0.05 was considered significant.

The join-point regression model was used to test the trend of reported incidence from 2005 to 2017. In the model, trends were described by annual percentage changes (APC). The estimation of APC was by fitting a simple linear logarithmic regression model. The Z test was used to evaluate whether APC was significantly different from 0. Non-significant (*P* ≥ 0.05) APC were described as stable, while significant (*P* < 0.05) positive or negative APC were described as increasing or decreasing.

The analysis was performed using SPSS software (version 20.0) and Joinpoint (version 4.8.0.1).

## Results

### Characteristics of patients

From 2005 to 2017, a total of 239,707 new active PTB cases were reported in seven selected cities in Shandong Province. The total number of elderly patients was 77,192 (32.2%), and the average age was 69.8 years. Among them, 59,284(76.80%) were male patients, Han nationality accounted for 99.87%, rural patients accounted for 91.06%, 39,958 cases (51.76%) were positive for sputum smears.

Compared with the non-elderly PTB, the proportion of men (OR 1.688, 95% CI 1.656–1.722), rural population (OR 3.411, 95% CI 3.320–3.505) and bacteriologically confirmed PTB (OR 1.213, 95%CI 1.193–1.234) in the elderly PTB cases was higher. (Table 1).

**Table 1:** Sociodemographic and clinical characteristics of the elderly and non-elderly PTB patients, Shandong Province, China, 2005–2017

Characteristics	Age ≥ 60 y, no. (%), *n* = 77,192	Age < 60 y, no. (%), *n* = 162,515	Total OR (95% CI), *n* = 239,707	*p* value
Total	77,192(32.20)	162,515(67.80)	239,707	<0.001
Sex				
Male	59,284(76.80)	107,624(66.22)	1.688(1.656–1.722)	<0.001
Female	17,908(23.20)	54,891(33.78)		
Stratum				
Rural	70,288(91.06)	121,729(74.90)	3.411(3.320–3.505)	<0.001
Urban	6904(8.94)	40,786(25.10)		
Ethnic group				
Han	77,092(99.87)	161,786(99.55)	3.474(2.818–4.282)	<0.001
Other	100(0.13)	729(0.45)		
Patients type				
Bacteriologically confirmed PTB	39,958(51.76)	76,280(46.94)	1.213(1.193–1.234)	<0.001
Clinically diagnosed PTB	37,234(48.24)	86,235(53.06)		
Geographical location				
Jinan	6336(8.21)	15,354(9.45)		
Yantai	7213(9.34)	20,914(12.87)		
Weifang	8281(10.73)	23,486(14.45)		
Linyi	23,078(29.90)	40,330(24.82)		
Dezhou	8837(11.45)	16,771(10.32)		
Liaocheng	12,352(16.0)	22,639(13.93)		
Jining	11,095(14.37)	23,021(14.17)		<0.001

*OR* Odds ratio, *PTB* Pulmonary tuberculosis

### Total and annul incidence rate

The overall reported incidence of the elderly was 68.84/100,000, and the reported incidence of PTB in the elderly was significantly higher than that of non-elderly people. From 2005 to 2017, the annual reported incidence of newly active PTB (78.79–51.31/100,000) in the elderly dropped by about 35%, and the reported incidence was the highest in 2007, at 88.75/100,000. The reported incidence of bacteriologically confirmed PTB in elderly patients (67.32–13.13/100,000) decreased by approximately 81%. (Table 2).

**Table 2:** Incidence of pulmonary tuberculosis in Shandong, China, 2005–2017

	Incidence per 100,000 population													Change^a^(%)
	2005	2006	2007	2008	2009	2010	2011	2012	2013	2014	2015	2016	2017	2005–2017
**Total**	33.56	37.43	39.74	41.83	39.78	38.41	35.59	36.28	35.47	33.57	31.16	28.48	27.50	−18.06%
**Total(≥60)**	78.79	81.36	88.75	88.30	77.63	74.43	67.23	71.04	65.96	61.14	58.77	52.87	51.31	−34.88%
**Total(<60)**	26.72	30.26	31.43	33.98	33.11	31.90	29.54	29.31	28.97	27.43	24.66	22.50	21.64	−19.01%
*P* value	<0.01	<0.01	<0.01	<0.01	<0.01	<0.01	<0.01	<0.01	<0.01	<0.01	<0.01	<0.01	<0.01	
Sex**(≥60)**														
Male	122.05	124.83	136.02	134.83	117.17	114.12	101.50	107.40	98.76	91.51	88.82	78.25	75.41	−38.21%
Female	34.32	36.76	40.29	40.66	37.06	33.71	31.94	33.35	32.00	29.62	27.61	26.69	26.68	−22.26%
Sex**(<60)**														
Male	34.28	38.8	40.45	43.54	43.09	41.84	38.53	38.41	37.96	35.99	32.77	29.78	28.88	−15.75%
Female	18.94	21.5	22.16	24.17	22.85	21.67	20.28	19.9	19.68	18.55	16.24	14.95	14.14	−25.34%
*P* value	<0.01	<0.01	<0.01	<0.01	<0.01	<0.01	<0.01	<0.01	<0.01	<0.01	<0.01	<0.01	<0.01	
Patients type**(≥60)**														
Bacteriologically confirmed PTB	67.32	65.67	63.5	58.84	51.44	47.88	35.73	28.42	24.75	16.83	15.43	14.57	13.13	−80.5%
Clinically diagnosed PTB	11.48	15.71	25.29	29.5	26.21	26.6	31.51	42.54	41.16	44.28	43.34	38.41	38.42	234.67%
Patients type**(<60)**														
Bacteriologically confirmed PTB	21.24	22.9	20.42	19.75	19.15	17.89	13.51	10.22	9.14	6.11	5.05	4.66	4.12	−80.6%
Clinically diagnosed PTB	5.48	7.36	11.01	14.23	13.95	14.01	16.03	19.09	19.83	21.32	19.61	17.85	17.52	219.71%
*P* value	<0.01	<0.01	<0.01	<0.01	<0.01	<0.01	<0.01	<0.01	<0.01	<0.01	<0.01	<0.01	<0.01	

*PTB* Pulmonary tuberculosis

^a^The % changes were calculated as follows: (incidence in 2017 – incidence in 2005)/incidence in 2005

### Temporal trends and join-point regression model

The join-point regression model showed that the overall reported incidence of PTB in the elderly remained at a stable level from 2005 to 2007, with a low annual increase rate (APC = 6.9, *P* = 0.3); it decreased significantly from 2007 to 2017 (APC = -5.3, *P* < 0.05). The reported incidence of bacteriologically confirmed PTB among elderly patients declined rapidly from 2005 to 2014. It decreased by 7.2% per year from 2005 to 2010 (*P* < 0.05), and it decreased by 22.6% from 2010 to 2014 (*P* < 0.05). It remained stable from 2014 to 2017 and the annual decline rate was low (APC = -9.0, *P* = 0.1).

**Fig. 1:**
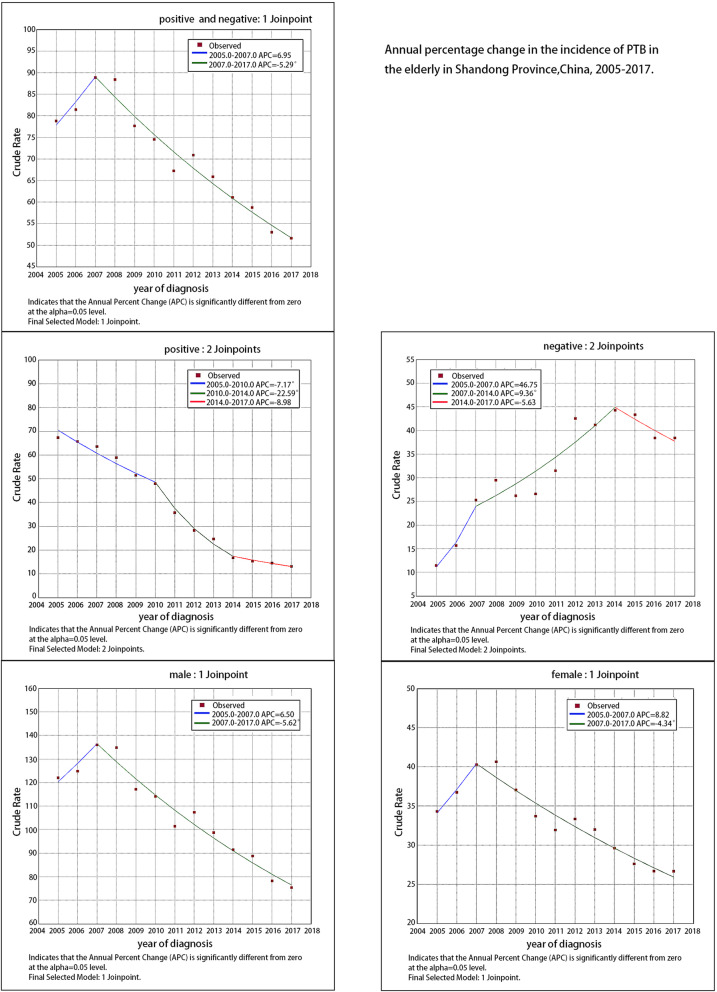
Annual percentage change in the reported incidence of PTB in the elderly in Shandong Province, China, 2005–2017. PTB = pulmonary tuberculosis

The reported incidence of clinically diagnosed PTB among elderly patients from 2005 to 2017 (11.48–38.42/100,000) increased by about 235%. It remained stable from 2005 to 2007, with a low annual rate of increase (APC = 46.8, *P* = 0.2), it rose significantly from 2007 to 2014 (APC = 9.4, *P*<0.05), and remained stable from 2014 to 2017 (APC = -5.6, *P* = 0.4). (Table 2**,** Table 3**,** Fig. 1).

**Table 3:** Annual percentage change in incidence of pulmonary tuberculosis in Shandong, China, 2005–2017

	Period	Trend	APC (95% CI)	*P*-value
**Total(≥60)**	2005–2007	Stable	6.9(−5.7, 21.3)	0.3
	2007–2017	Decrease	−5.3(−6.2,-4.4)	<0.05
Sex				
Male	2005–2007	Stable	6.5(−7.1,22.1)	0.3
	2007–2017	Decrease	−5.6(−6.6,-4.6)	<0.05
Female	2005–2007	Stable	8.8(−5.0,24.6)	0.2
	2007–2017	Decrease	−4.3(− 5.3,-3.4)	<0.05
Patients type				
Bacteriologically confirmed PTB	2005–2010	Decrease	−7.2(−9.8,-4.4)	<0.05
	2010–2014	Decrease	−22.6(−28.7,-15.9)	<0.05
	2014–2017	Stable	−9.0(−18.4,1.5)	0.1
Clinically diagnosed PTB	2005–2007	Stable	46.8(−30.2208.4)	0.2
	2007–2014	Increase	9.4(1.8,17.5)	<0.05
	2014–2017	Stable	−5.6(−20.4,11.9)	0.4
**Total(<60)**	2005–2008	Increase	7.8(2.9,13.0)	<0.05
	2008–2014	Decrease	−3.7(−5.6,-1.7)	<0.05
	2014–2017	Decrease	−8.0(−12.7,-3.1)	<0.05
Sex				
Male	2005–2008	Increase	8.1(2.9,13.5)	<0.05
	2008–2014	Decrease	−3.4(−5.4,-1.3)	<0.05
	2014–2017	Decrease	−7.7(−12.5,-2.6)	<0.05
Female	2005–2008	Increase	7.3(2.5,12.3)	<0.05
	2008–2014	Decrease	−4.4(−6.3,-2.5)	<0.05
	2014–2017	Decrease	−8.8(−13.5,-3.9)	<0.05
Patients type				
Bacteriologically confirmed PTB	2005–2010	Decrease	−4.9(−8.4,-1.3)	<0.05
	2010–2017	Decrease	−20.7(−23.6,-17.6)	<0.05
Clinically diagnosed PTB	2005–2008	Increase	33.2(18.4,49.9)	<0.05
	2008–2014	Increase	7.8(4.0,11.7)	<0.05
	2014–2017	Stable	−6.6(−13.4,0.7)	0.1

*APC* Annual percent change, *PTB* Pulmonary tuberculosis

The trend of male and female reported incidence among elderly patients was consistent with the overall trend, which was stable from 2005 to 2007 and significantly decreased from 2007 to 2017 (*P* < 0.05). The proportion of male patients in elderly patients was significantly greater than that of female patients. (Tables 2 and 3).

### TB cases by age group and aging trend

**Fig. 2:**
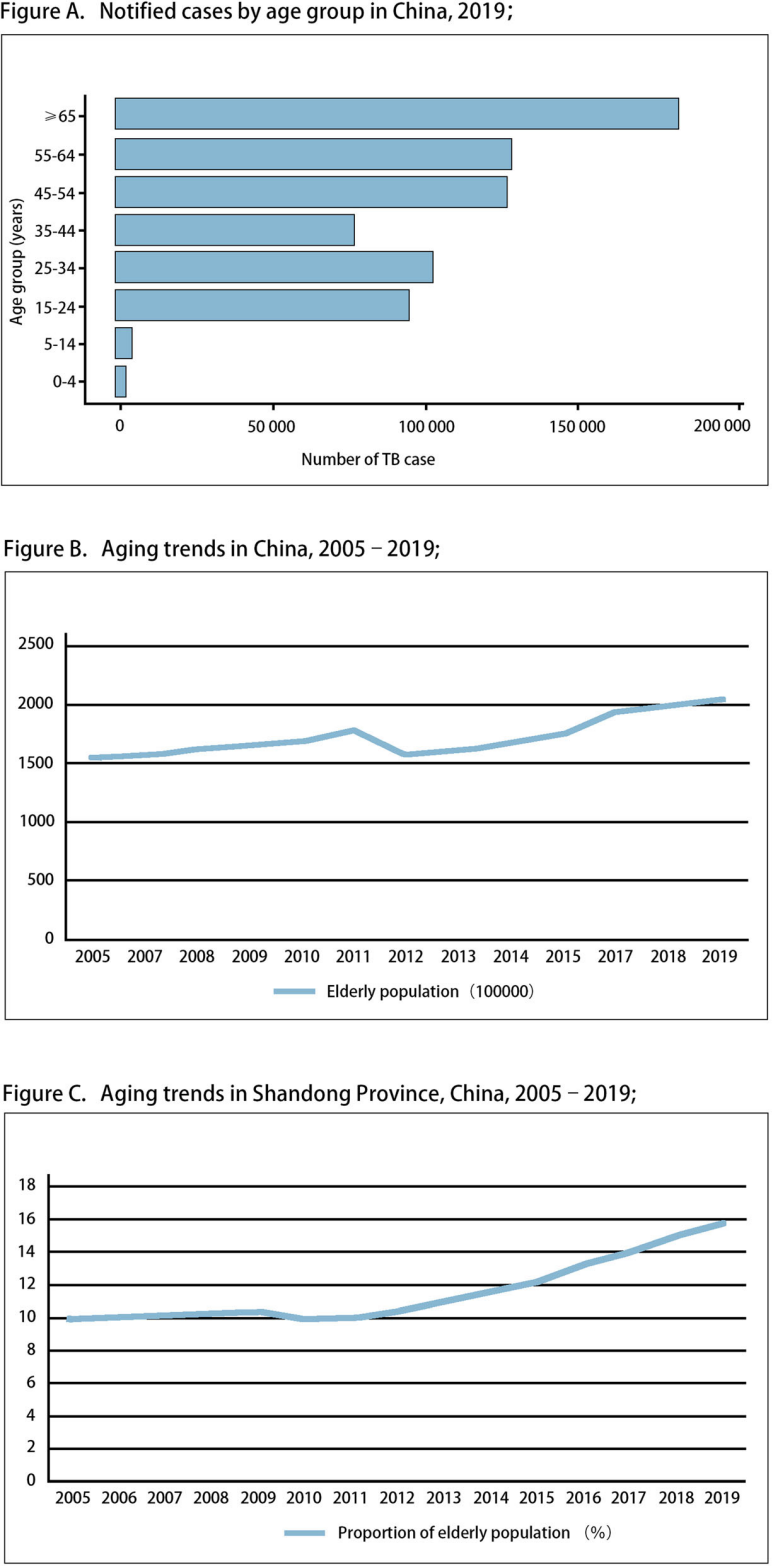
**a**, Global estimates of TB incidence, 2019; **b**, Aging trends in China, 2005–2019; **c**, Aging trends in Shandong Province, China, 2005–2019; TB = tuberculosis

The Global Tuberculosis Report grouped the reported TB cases by age, 2019 [3]. (Fig. 2a).

From 2005 to 2019, the aging population of China and China’s Shandong Province had shown an upward trend year by year [10]. (Fig. 2b, c).

## Discussion

As the aging of population intensifies, investigating and understanding the prevalence of PTB in the elderly has the important meaning for more effective prevention and control of tuberculosis. This study analyzes the clinical and epidemiological characteristics of PTB in Shandong Province, China. The main findings are: 1) The reported incidence of newly active PTB in the elderly is significantly higher than that of non-elderly people; 2) compared with non-elderly patients, newly active elderly PTB patients account for a greater proportion of male cases, rural population cases and bacteriologically confirmed PTB cases; 3) The reported incidence of newly active PTB in the elderly and the reported incidence of bacteriologically confirmed PTB both decreased significantly, and the reported incidence of clinically diagnosed PTB in elderly cases increased significantly.

In this study, a total of 239,707 cases of newly active PTB were reported in seven cities in Shandong Province from 2005 to 2017. Its annual reported incidence rate was 35.21 per 100,000, which was lower than the global data in 2018 [1], and also lower than many other reported incidence levels in China [11–17]. The reported incidence is at a low level nationwide. This shows that the prevention and treatment of tuberculosis in China’s Shandong Province has achieved good effects.

In this study, the average annual reported incidence of newly active PTB in the elderly was much higher than that of the non-elderly. Similarly, tuberculosis were also more common among the elderly in many countries such as the United States, the United Kingdom, Japan, and other East and Southeast Asian countries [18–20]. The tuberculosis incidence rate in Africa peaked among population aged 25 to 44 [20]. The age distribution of tuberculosis incidence in Africa was different from our study. This was due to its high proportion of tuberculosis cases co-infected with HIV among young people in Africa [1, 18]. It was well-known that HIV-infected people had a 19 times higher risk of tuberculosis than normal people [1, 18]. Actually, most previous studies [18, 20] are consistent with our conclusions, which suggest that the increased incidence of newly active PTB due to aging is a common problem in TB control. After *Mycobacterium tuberculosis* infection, there are two cases of the onset: the onset of new infection and the activation of latent infection. Changes in immune function among elderly were considered to be an important risk factor for the increased susceptibility to tuberculosis and the reactivation of latent tuberculosis infection [18, 19, 21]. The potential mechanisms of impaired immune system function among aging population included various DNA damage, protein misfolding, and decreased cell function at the cellular and molecular level [22, 23]. Research indicated that the lungs became more inflammatory with age on level of individuals. These all increased the risk of tuberculosis infection in the elderly [21]. The risk of latent tuberculosis infection accumulated throughout life [24], so its incidence was higher among older people [25]. In addition, aging was also a major risk factor for some human diseases, such as cancer, diabetes, cardiovascular disease and neurodegenerative diseases [23], which increased the risk of PTB. In sum, population aging may lead to a high incidence of newly active PTB.

PTB was divided into bacteriologically confirmed and clinically diagnosed PTB [26]. TB patients plays an important role as the infection source. Bacteriologically confirmed PTB was more contagious, and might cause PTB outbreaks in some areas [27–31]. In this study, the proportion of bacteriologically confirmed cases in the elderly was higher than that of the non-elderly people (OR 1.213, *P* < 0.001). Some research showed that the incidence of tuberculosis in men was higher than that in women [27, 32]. Our study showed that compared with women, aging may have a greater impact on increasing the reported incidence of PTB in men (OR 1.688, *P* < 0.001). This phenomenon can be explained by the physiological differences between the sexes, social and cultural differences such as smoking, alcohol, drug abuse and other behavioral risk factors, as well as social network patterns that affect the source of infection [18]. Furthermore, this study showed that aging might increase the incidence of newly active PTB in rural population more than urban population (OR 3.411, *P* < 0.001). Poor economic conditions, urban-rural differences in life and production methods, and low awareness of tuberculosis might be the reasons for the high incidence of tuberculosis in rural areas [33–35]. The aging population may exacerbate the epidemic of PTB. Therefore, the above results suggest that elderly men in rural areas may be the priority of tuberculosis prevention and control work in future.

China’s strict regulations and measures for tuberculosis, including routine infectious disease reporting system, directly-observed treatment strategy (DOTS) [36, 37], and some non-tuberculous specific interventions such as improving living standards and improving the environment [38], were the main reasons for the decline of the overall reported incidence of PTB in Shandong. In this study, the overall reported incidence of PTB and the reported incidence of bacteriologically confirmed PTB decreased from 2007 (*P* < 0.05). Pharmacological methods alone were not enough to treat tuberculosis, and social determinants of health must also take into account. This could really improve the burden of tuberculosis [39]. Therefore, some measures taken by the Shandong Provincial Government around 2007 should explain why the trend in the reported incidence of PTB began to decline in 2007. These measures included the implementation of Shandong Province’s policy to completely abolish agricultural taxes, the implementation of Shandong’s rural residents’ minimum living security system, and further measures against environmental pollution, such as the province’s pollution source survey conducted in early 2008.

In this study, the reported incidence of clinically diagnosed PTB increased by 234.67%, which increased sharply from 2005 to 2014, similar to the results of other studies [37]. This suggests that the burden of PTB has gradually changed from bacteriologically confirmed cases to clinically diagnosed cases. The sensitivity of TB diagnosis is low depending only on symptoms, chest radiography and AFB sputum smear [40]. Therefore, to improve the diagnosis of clinically diagnosed PTB, many countries and organizations, including China, had carried out specific work, such as the development and use of TB antibody test, interferon-γ release assay, T cell detection, HRCT, bronchoscopy and other diagnostic methods [41–43]. This may be the main reason why the reported incidence of clinically diagnosed PTB increased. In addition, the role of ultrasound in the diagnosis of tuberculosis should also be concerned. Ultrasound was an effective diagnostic tool in detecting signs of extra-pulmonary tuberculosis. For example, ultrasound could be used to detect tuberculosis-related effusion, residual pleural thickening, mediastinal lymphadenopathy, and transthoracic biopsy guidance [44, 45]. Therefore, ultrasound can assist in the diagnosis of PTB with extra-pulmonary tuberculosis. Clinically diagnosed PTB could also cause the spread of tuberculosis [46], and was more difficult to diagnose [47, 48]. Therefore, as the burden of disease shifts, it is very important to diagnose and treat the patients with presumptive clinically diagnosed PTB as soon as possible.

The limitations of this study were as follows: First, studies had shown that tuberculosis recurrence or activation of latent infections would be a major factor of TB patients morbidity and mortality in the future [38]. Aging might increase the recurrence of tuberculosis [49]. However, our study only included newly cases of PTB. Therefore, further researches on recurrent PTB cases could be considered in near future. Second, since only one province in eastern China was examined, regional differences may limit the generalizability of the results.

## Conclusion

Population aging was an important risk factor for the increased reported incidence of total newly active PTB. The main burden of PTB in Shandong were shifting to males, rural population, and clinically diagnosed cases. Therefore, elderly men in rural areas are probably going to be the main focus of tuberculosis prevention and control. The application and development of rapid and accurate diagnostic methods for clinically diagnosed PTB is vitally important for further TB control.

## Data Availability

The datasets used and/or analysed during the current study are available from the corresponding author on reasonable request.

## Abbreviations

PTB: Pulmonary tuberculosis
TB: Tuberculosis
OR: Odds ratio
CI: Confidence interval
WHO: The World Health Organization
CDC: Center for Disease Control and Prevention
AFB: Acid-fast bacilli
APC: Annual percentage changes
DOTS: Directly-observed treatment strategy
